# The Sense of Taste and Effect of Plates Worn in the Mouth

**Published:** 1852-10

**Authors:** W. H. Dwinelle


### SELECTED ARTICLES.

### ARTICLE XIII.

### *The Sense of Taste and Effect of Plates worn in the Mouth.*

By W. H. Dwinelle, M. D.

Nature in her wise economy, has supplied us with various organs and appliances, each of which has a legitimate office for a specific purpose; and although the spirit from its inner temple, may so marshal a part, or all of these individual agencies, so as to produce results collateral to themselves; yet each has an individuality, and each has an office and a power assigned to it, which cannot be performed or usurped by another.

With our eyes we are enabled to see, with our ears to hear; and with our tongues to taste; and over all of these, and every other organ, and part of the body, is given the ability to feel, —the *mind*, however, alone sees, hears, feels, &c., &c., and these are its instrumentalities, these are the delicate media, and these the ingenious mechanisms, through which she receives her impressions.

There is a concert and harmony of action, in the operation of all these appliances, and though one organ may indirectly assist another—or directly, as in the case of those of taste and smell—yet it never takes upon itself duties and offices, extraneous to its own character, or the intention of nature.

The eye never takes upon itself the duties of the ear; the ear never meddles with optics; nor does the organ of smell ever push its nose into the affairs of either. We never attempt to perform the office of smelling with our tongue, nor can we taste with our *rugæ!* And here, Mr. Editor, we find ourselves introduced at once to the very "head and front" of the matter, in regard to which we sat down to write. We will at once press forward our question. *Do we ever taste with our rugæ?* We answer, most emphatically, no, under no possible combination of circumstances, or course of discipline, could one individual ruga be made to answer as the poorest substitute for the tongue; for the simple reason, that the rugæ were never designed for the purpose, and are not supplied with nerves of taste as is the tongue. In common with the latter organ, it has the sense of feeling; it has other offices and uses, but tasting is not one of them.

We have intimated that the rugæ have offices and uses peculiar to themselves. By their ability to feel, they aid the tongue in locating food in masticating; they are material aids in articulating, especially the dental sounds; but their chief and most practical office and use is, to answer the purpose of a macerating board for spreading and displaying the food, so that the greatest possible surface may be exposed to the nerves of the tongue. Since the tongue can best perform its office when brought in direct contact with some opposing or neighboring part of the mouth, the rugæ, by their complete antagonism with the tongue, furnish it with most valuable assistance. Some of the nerves of taste are distributed around the fauces, uvula and posterior portions of the vellum, but none of them ever have, or ever will, so far encroach upon the palatine ridge, as to supply the rugæ with offices not their own. And yet, it is a popular error, that "*we taste with the roof of the mouth!*"

The number, character and origin of the nerves, employed and distributed about the organ of taste, still remains a matter of controversy; yet all, I believe, agree that the *glosso pharyngeal*, a branch of the eighth pair of nerves, and terminating upon the mucous surfaces of the fauces, and back part of the tongue; and the *lingual branch* of the fifth pair which mostly supply the upper part of the tongue, and is copiously distributed to the papillæ near the tip—constitute the chief nerves by which impressions of taste are realized in the great sensorium.

Raspail says, that "for the tongue to perceive savors, it must be placed in communication with some other part, or even with a foreign substance, by means of the vapid body. Thus, if we dip the end of the tongue in a vapid liquid, we experience merely a sensation of touch, but none of taste. But if we now apply the end of the tongue to a silver spoon, the perception of taste, he says, is immediately developed. So, if we moisten with a little syrup, the lips, gums, teeth, or palate, no perception of taste will be excited in these parts; but if we now apply the tip or edges of the tongue to the moistened part, we shall immediately perceive the taste of the syrup. Hence, Raspail considers the tongue alone as the proper seat of the taste; but for the production of the sensation, two other factors are necessary, viz. a vapid substance, and a third body, with which the tongue is brought into communication by means of the former. The process he regards as a galvanic one; the tongue representing the positive pole, the palate, cheeks, lips, teeth, &c., the negative, and the vapid body, which must be in a fluid state, as completing the circle. Raspail illustrates this view by the well known experiment of placing the end of the tongue between two pieces of coin, one of silver, the other of copper or gold. When in this position, if the edges of the two coins are brought together, an acid taste is perceived at the instant of contact, but disappears the moment this is interrupted. The perception of taste in all other cases, he maintains, is the result of a similar mechanism, and the tongue, by itself, although the organ of taste, is incapable of tasting anything."

We do not entirely endorse Raspail's theory, for the tongue is capable of tasting, though imperfectly, without being brought in contact with opposing substances. Yet admitting his hypothesis to be correct, it only supports our theory, for the plate of gold is in a negative electric state of itself, besides, from its immediate proximity to the rugæ, it is the best possible conductor to it.

It may be contended by those who would advocate the gustability of the rugæ, that as the tongue cannot perfectly taste until the vapid body has by its aid been brought in contact with it, we are not justified in concluding that one organ more than the other tastes the intervening substance. A single experiment will at once decide the superiority of the tongue. Moisten a piece of thick writing paper; on one side of it sprinkle a few grains of sugar; and on the other, a few grains of tartaric acid, in such a manner that it will not fall off. Carefully apply this, acid side up, to the rugæ; now bring the tongue in contact with the paper, and the sweet taste of the sugar will be recognized, while the acid is nowhere perceptible. Now remove the paper, rinse the mouth, and apply it as before, sugar side up—the tongue detects the acid, and the rugæ has tasted neither.

"That was an excellent set of teeth you constructed for my wife, Doctor—never saw anything more beautiful; the shape, character, and tinting of the teeth so exactly correspond to her original ones, I could almost have sworn you had had the natural set I first became acquainted with many years ago before you, to copy from; and this is not all, you have restored the contour of her face to its original character and beauty. In place of that narrowness and sunken expression about the upper lip, you have, by an additional application of your art, rounded it out to the same breadth and fullness, and given the same pleasing and natural expression which she wore long before you saw her, the same expression which constitutes one of the greatest charms of her portrait by Sully, painted when she was young, but which, unfortunately, some time ago, I had felt obliged to send away into the garret, the contrast between the two was so great; now, they will do to live together again; it shall hang in its old place in the parlor.

You have elevated your art to something far more than mere dentistry, something more than to supply the loss of the teeth; you restore the lost expression, the lost peculiar smile, the lost familiar and family likeness; you give us back our youth, and restore to us our friends as they were in days of yore—indeed, you seem to have pressed many arts into the service of your own; like a physiologist, you seem to have had an eye to comparative anatomy, and like an artist, to have studied form, color, and harmony. But, Doctor, there is one great drawback to your success in the case of Mrs. B.; she masticates readily with her teeth, their adaptation is firm yet easy, but they have ruined her taste, Doctor, while her mouth is covered with the plate, she tastes nothing at all, every delicacy and every variety of food is alike tasteless to her; cannot you devise a remedy, Doctor?"

Many have listened to just such a combination of compliments and complaints as the above, and, unfortunately, many to remedy the supposed evil, have gone back to narrow plates and springs, so that the rugæ might be left free to *taste!*

Learned dissertations have been written, and ingenious contrivances invented, for the purpose of leaving the rugæ free to exercise a function they do not possess.

A few simple experiments are always sufficient to convince the class of patients under consideration, not only that they do taste as well as before, while the plate is in their mouth, but also of the manner into which they have fallen into their error.

Let them open the mouth and place their tongue in such a position that it will not come in contact with the rugæ, now let them place successively different articles of food, condiments, &c., &c., upon the rugæ; sand and sugar, acid and honey, alike make the same impression, and that impression is only through the nerves of feeling.

When a broad plate is first placed in a patient's mouth, the rugæ being covered thereby, no longer feels the presence of the tongue; the tongue has been accustomed to feel the rugæ, and the rugæ to echo back the feeling; now this antagonism and reciprocity is broken up by the barrier between, creating a difference and confusion of feeling, which is easily confounded into a loss of taste. Unfortunately, oftentimes, poisonous oxyds from impure plates, vitiating the secretions of the mouth, favor the delusion. We need no better evidence of its being a delusion, than the fact that all intelligent patients who have gone through with the above experiments, have invariably found their *taste restored to them!*

The formation of the rugæ is reproduced upon the plates when struck up in a proper manner, rendering them in all respects, save their inability to feel, equal to the natural rugæ underneath.

For the highest degree of permanency, comfort, and practicability in full upper sets, we need strong, thick, broad plates, covering all, or nearly all of the hard palate; if such plates are of a proper degree of purity, and are aided by care and cleanliness in their use, no reasonable objection can be urged against them.

